# High-Minority Nursing Homes Disproportionately Affected by COVID-19 Deaths

**DOI:** 10.3389/fpubh.2021.606364

**Published:** 2021-03-22

**Authors:** Robert Weech-Maldonado, Justin Lord, Ganisher Davlyatov, Akbar Ghiasi, Gregory Orewa

**Affiliations:** ^1^University of Alabama at Birmingham, Birmingham, AL, United States; ^2^Louisiana State University in Shreveport, Shreveport, LA, United States; ^3^The University of Oklahoma Health Sciences Center, Oklahoma City, OK, United States; ^4^University of the Incarnate Word, San Antonio, TX, United States

**Keywords:** COVID-19, mortality, nursing homes, disparities, race/ethnicity

## Abstract

Racial/ethnic disparities in healthcare have been highlighted by the recent COVID-19 pandemic. Using the Centers for Medicare and Medicaid Services' Nursing Home COVID-19 Public File, this study examined the relationship between nursing home racial/ethnic mix and COVID-19 resident mortality. As of October 25, 2020, high minority nursing homes reported 6.5 COVID-19 deaths as compared to 2.6 deaths for nursing homes that had no racial/ethnic minorities. After controlling for interstate differences, facility-level resident characteristics, resource availability, and organizational characteristics, high-minority nursing homes had 61% more COVID-19 deaths [Incidence Rate Ratio (IRR) = 1.61; *p* < 0.001] as compared to nursing facilities with no minorities. From a policy perspective, nursing homes, that serve primarily minority populations, may need additional resources, such as, funding for staffing and personal protective equipment in the face of the pandemic. The COVID-19 pandemic has sharpened the focus on healthcare disparities and societal inequalities in the delivery of long-term care.

## Introduction

COVID-19 is disproportionately affecting older individuals and those with underlying chronic health care conditions. The congregate nature of nursing homes and the average acuity of residents place them at higher risk of serious complications due to COVID-19, including death. More than 40% of COVID-19 deaths have been attributed to nursing home residents (1). Racial/ethnic disparities in nursing homes have been highlighted by the recent COVID-19 pandemic (2). Between 1999 and 2008, the number of older Hispanics residents in nursing homes grew by 55%; the number of Black residents increased 11%, while the number of White nursing home residents declined 10% (3). As Whites sought long-term care outside of the nursing home, Hispanics and Blacks increased their utilization of nursing homes (4). Blacks now account for 15% of all nursing home residents, Hispanics 6%, and Whites 79%. This has implications for health inequity. Minorities on average receive care from relatively lower quality providers and have worse health outcomes, which may increase the risk of mortality as it relates to COVID-19 (5).

The existence of disparate levels of care were identified by Mor and colleagues (6) in the seminal paper “Driven to Tiers: Socioeconomic and Racial Disparities in the Quality of Nursing Home Care” (6). Mor and colleagues found that across the United States 40% of Black residents, but only 9% of Whites, resided in low-tiered nursing home facilities (6, 7). Low-tiered nursing homes typically have a larger minority census; worse quality; more serious deficiencies; sicker residents; lower levels of staffing; high Medicaid payer-mix; and greater financial vulnerability as compared to the high performing organizations (6). The issue of de facto racial segregation of health care facilities has been notated by researchers and advocates (8). As such, concerns have been raised that nursing homes will become more segregated as a disproportionate percentage of minority residents seem to be relegated to low performing nursing homes, which may exasperate health disparities (3, 9). Recent COVID-19 outbreaks and deaths in nursing homes exemplify the racial/ethnic disparities in nursing homes (10). As of November 15, 2020, nursing homes in every state had experienced at least one COVID-19 death, resulting in over 69,872 nursing home related deaths (11).

This pandemic is still evolving. As such the field of literature about COVID in nursing homes is as well. Articles have explored the nursing home characteristics and staffing levels associated with COVID-19 cases (12, 13); racial/ethnic disparities in nursing homes' COVID-19 infection and deaths (10); and how the nursing home crisis may have been averted through changes in policy (14). This paper brings a unique contribution to this ever-growing field of literature. First, it uses the national Centers for Medicare and Medicaid Services' COVID-19 Public File through October 25th, 2020 to explore racial/ethnic disparities in nursing home mortality. Second, after controlling for resident characteristics, this study explores how resource availability and other organizational characteristics may affect nursing home racial/ethnic disparities in COVID-19 mortality. More specifically, this study examines how nursing homes with a high proportion of Black and Hispanic residents differed from nursing homes with no minority residents, low, or medium proportion of minority residents.

According to Resource Dependency Theory (RDT), the key to organizational performance is “the ability to acquire and maintain resources” (15). RDT suggests that organizations engage in exchange relationships with its environment, to acquire resources in order to function. Organizational factors can influence an organization's level of power in an environment, which in turn, will impact the ability of the organization to gain necessary resources for survivability. Resources are the inputs that organizations need to provide quality services. In this case having adequate levels of resources may have helped organizations better prepare and deal with the coronavirus. This analysis will provide some insights into the organizational and community factors associated with nursing homes who have been hit the hardest by COVID-19. Given the evolving nature of this pandemic, these findings may help policy-makers better understand the factors that place residents at greater risk of death due to COVID-19.

## Methods

### Data

This study utilized three secondary data sets: CMS Nursing Home COVID-19 Public File as of October 25, 2020, Brown University's LTCFocus, and Robert Graham Center's Social Deprivation Index. The CMS Nursing Home COVID-19 Public File includes data from the Centers for Disease Control and Prevention's (CDC) National Healthcare Safety Network. This is the first national data set to report cumulative COVID-19 related data retrospectively back to January 1, 2020. LTCFocus data provides nursing home organizational, demographic, quality, and market information. All of the variables with the exception of COVID-19 mortality and the county-level Social Deprivation Index came from LTCFocus. The Robert Graham Center contains data on the Social Deprivation Index, calculated based on socioeconomic and demographic characteristics of the nursing home county.

### Sample

The study sample consisted of all US nursing homes included in the CMS Nursing Home COVID-19 Public File, or 15,382 nursing homes, which mirrors the national census of facilities. After merging with the various secondary datasets, the study had 12,914 nursing homes in the final analytic sample. The original nursing home sample has 15,392 observations. There were no significant differences in the organizational, resource availability, or other control variables in the sample population and the full census.

### Variables

#### Dependent Variable: COVID-19 Mortality

The dependent variable was comprised of COVID-19 deaths per nursing home facility. The number of reported COVID-19 related deaths was calculated from January 1, 2020 to October 25, 2020 and came from the CMS Nursing Home COVID-19 Public File.

#### Independent Variables

The main independent categorical variable represented the proportion of racial/ethnic minority residents (proportion of Black and Hispanic residents): no minorities, low proportion of minorities; medium proportion of minorities; and high proportion of minorities. The reference group was nursing homes who reported no minorities. Nursing homes with 1% or higher of minorities were classified into three groups based on tertiles: low proportion of minority residents (1–13.3%), hereinafter, low-minority nursing homes; medium proportion of minority residents (<13.3– ≤ 30.3%), hereinafter medium-minority nursing homes; and high proportion of minority residents (>30.3%), hereinafter, high-minority nursing homes. This study only included Black/Hispanic as minorities. Previous research has found differences in the quality of care for Black and Hispanic residents as compared to other racial/ethnic groups (16). The remaining percentage of “other” race/ethnicity was only 6% and was included with the no-minority group.

Resource availability and other organizational characteristics may affect racial/ethnic disparities in COVID-19 mortality. Resource availability included nursing homes' payer-mix (percent of Medicare and Medicaid); occupancy rate; the county-level Social Deprivation Index (SDI); and nursing home location (metro and non-metro). *Payer mix* identifies the proportion of the facilities residents who are on Medicaid and Medicare. *Occupancy rate* is the percentage of occupied nursing home beds. As the occupancy rate decreases, nursing homes will have less revenue, which ultimately can impact the ability of the nursing home to provide quality care. However, in the case of COVID-19, with greater occupancy there may be higher infection rates. *Social Deprivation Index* is a composite measure of socio-economic factors, that includes items, such as, percent living in poverty, <12 years of schooling, crowding, no car, non-employed, renter occupied, and single parent households at the country level, which was derived from the Robert Graham Center (17). *Location* (metro and non-metro) was derived using the Rural-Urban Continuum Codes (RUCC) and was included to capture factors associated with geographic location.

Other organizational characteristics captured factors, such as, nursing home for-profit status (ownership), chain affiliation, and self-reported nursing, clinical, aides, and other staff shortages. *Ownership* is a dichotomous variable that identifies whether a nursing home is for-profit (0 = not for-profit; 1 = for-profit). *Chain affiliation* reflects whether the nursing home is part of a chain (0 = free-standing; 1 = chain affiliated). Reported nursing, clinical, aides, and other staffing shortages were captured by the CMS Nursing Home COVID-19 Public File. Shortages of nursing staff, included registered nurse, licensed practical nurse, vocational nurse as reported by the provider. Shortage of clinical staff examined the availability of physician, physician assistant, advanced practice nurse as reported by the provider. Shortage of aides was conceptualized as a shortage of certified nursing assistant, nurse aide, medication aide, and medication technician as reported by the provider. Shortage of other staff was described as staffing shortage of other staff or facility personnel, regardless of clinical responsibility or resident contact not included in the categories above (for example, environmental services) as reported by the provider. If nursing homes reported yes to any of these questions, it was reported that nursing homes had provider staffing shortages.

#### Control Variables

Control variables comprised of facility-level resident characteristics that may increase the risk of COVID-19 mortality: percent of females, percent of residents 65 years and older, percent of residents with congestive heart failure, hypertension, and obesity, and the average level of residents' acuity. *Percent of females* is the percent of all nursing home residents who were female. *Percent of individuals 65 and older* is the proportion of all residents who are 65 and older to the total nursing home population. *Percent of residents with congestive heart failure, hypertension, and obesity* are all underlying health conditions that increase the risk of health problems. *Acuity Index* is an average measure of the resident's level of care needed. This measure is based on the number of residents needing various levels of assistance with mobility, activities of daily living (ADL), special treatments, as well as, the proportion of residents that are bedfast, exhibit dementia and who require assistance with ambulation or transfers.

## Analysis

Bivariate statistics were conducted to examine nursing homes' characteristics as they related to proportion of minorities (high, medium, low, and no minorities). Multivariate regressions were used to model the relationship between COVID-19 deaths and the independent variables. Negative binomial regressions were used given the overdispersion, or the presence of greater variability than would be expected of the count dependent variable (number of COVID-19 deaths). The negative binomial coefficients are reported in incident-rate ratio (IRR) form. This study used four nested sequential models to examine the separate contributions of facility-level resident characteristics, resource availability, and other organizational characteristics to racial/ethnic disparities in COVID-19 deaths. In addition, size and interstate differences were controlled for using state fixed effects. A description of each model is listed below:

Model 1 analyzed the relationship between nursing home resident racial/ethnic mix, no minorities, low-minorities, medium-minorities, and high-minorities, and COVID-19 mortality. This model controlled for nursing home size and state fixed effects.
Model 2 included the variables from model 1, and in addition, controlled for facility-level resident characteristics, such as, percent of females, percent of residents 65 years and older, percent of residents with congestive heart failure, hypertension, and obesity, and the average level of residents' acuity.
Model 3 included the variables from model 2, and in addition, variables associated with resource availability, which was conceptualized as the nursing home's payer-mix (percent of Medicare and Medicaid), occupancy rate, the county-level Social Deprivation Index (SDI), and location (metro and non-metro).
Model 4 included variables from model 3, and in addition, other organizational characteristics comprised of nursing home for-profit status, chain affiliation, and reported provider nurse, clinical, aides, and other staffing shortages.

Multicollinearity was not a concern given that there were no correlations above 0.8, a typical threshold to establish collinearity, and no variance inflation factor (VIF) score exceeded 2.5 (18). Thus, all the variables were used in the multivariate analyses. All analyses were conducted using Stata 16, and statistical significance was established at *p* < 0.05.

## Results

**Figure 1:**
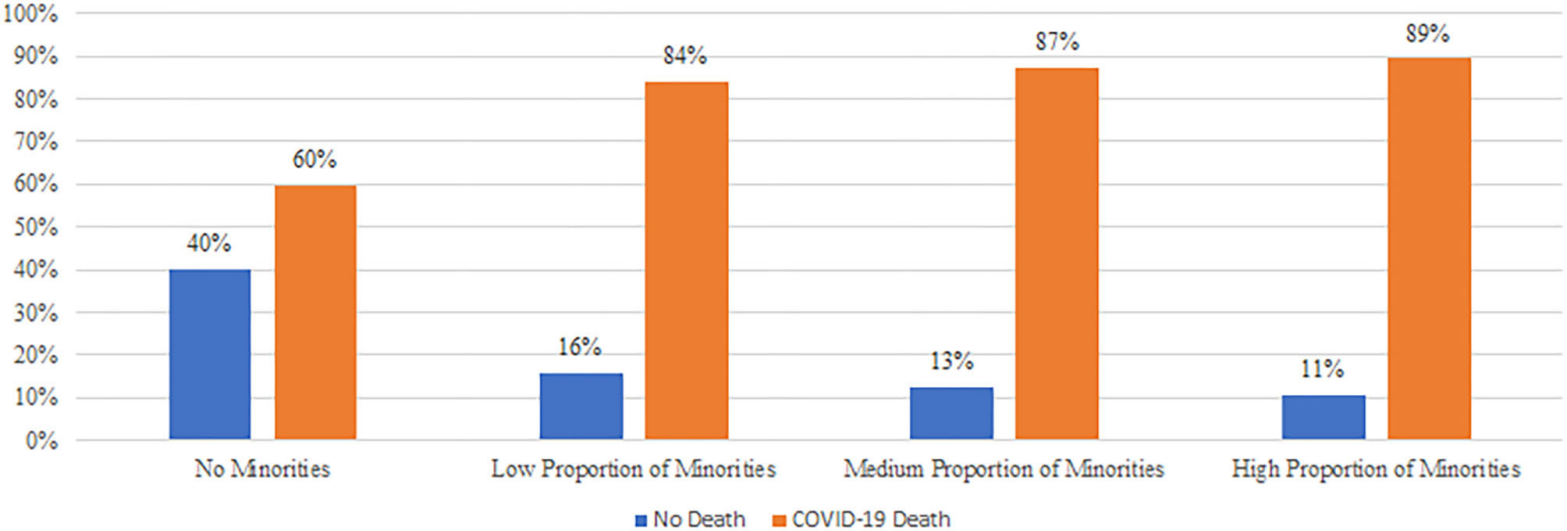
Nursing homes with COVID-19 death by resident racial/ethnic composition (*n* = 12,914). Source: Centers for medicare and medicaid services' nursing home COVID-19 public Blacks/Hispanics: Low proportion of minorities (0 < minority > 13.3); Medium proportion of minorities (13.3 < minority < 30.3); High proportion of minorities (30.3 < minority).

As of October 25, 2020, 89.4% of high-minority nursing homes reported at least one COVID-19 death as compared to 59.8% with nursing homes that had no racial/ethnic minorities (Figure 1). The bivariate analysis (Table 1) shows that nursing homes with racial/ethnic minorities experienced higher COVID-19 mortality than those with no-minorities, with high minority nursing home having the highest mortality (average = 6.5) and low minority the lowest mortality (average = 6). High-minority nursing homes had residents who tended to be younger, male, obese, with higher rates of hypertension, and worse acuity. When examining resource availability, high-minority nursing homes tended to have a higher Medicaid payer-mix and higher occupancy, and to be located in metro areas and communities with higher levels of social deprivation. Finally, high-minority nursing homes tended to be larger, for-profit, chain-affiliated, and reported less nurse, clinical, aides, and other staffing shortages.

**Table 1:** Bivariate statistics of the relationship between study variables and nursing home racial/ethnic mix (*N* = 12,914).

**Variables**	**No Minorities Mean (SD)**	Low Minorities^*^ (1–13.3%) Mean (SD)	Medium Minorities^*^ (<13.3–≤30.3%) Mean (SD)	**High Minorities (>30.3%) Mean (SD)**	***p*-value**
COVID-19 Death	2.66 (6.40)	6.00 (9.60)	6.01 (9.26)	6.50 (9.50)	0.001
**Resident characteristics**					
Female residents (%)	69.73 (14.19)	68.98 (9.79)	64.64 (11.21)	58.59 (12.13)	0.001
Residents 65 + (%)	94.07 (6.41)	91.79 (12.23)	85.24 (15.95)	77.46 (18.66)	0.001
Residents with Congestive Heart Failure (%)	15.42 (14.01)	19.41 (10.82)	16.56 (11.18)	14.66 (10.55)	0.001
Residents with Hypertension (%)	72.53 (16.60)	76.30 (12.43)	75.59 (14.07)	77.49 (12.85)	0.001
Residents who are Obese (%)	22.41 (14.86)	23.36 (10.33)	24.19 (10.51)	25.17 (9.73)	0.001
Acuity index	11.86 (1.40)	12.32 (1.11)	12.47 (1.30)	12.65 (1.69)	0.001
**Resource availability**					
Medicaid share (%)	57.17 (23.15)	53.26 (22.49)	63.09 (20.61)	69.79 (19.58)	0.001
Medicare share (%)	12.49 (12.79)	20.19 (15.76)	15.55 (13.19)	12.30 (10.60)	0.001
Social Deprivation Index	34.81 (23.56)	43.40 (24.84)	56.91 (25.51)	72.30 (23.22)	0.001
Location					0.001
Metro	3,412 (51.42)	1,802 (87.10)	1,722 (83.43)	1,811 (85.18)	
Non-metro	3,223 (48.58)	267 (12.90)	342 (16.57)	315(14.82)	
Occupancy (%)	80.89 (15.01)	82.88 (12.54)	81.88 (13.54)	83.48 (13.06)	0.001
**Organizational characteristics**					
**For profit status**					
No	2,688 (40.45)	505 (24.34)	351 (16.98)	331 (15.56)	0.001
Yes	3,957 (59.55)	1,570 (75.66)	1,716 (83.02)	1,796 (84.44)	
**Chain affiliation**					
No	2,961 (44.56)	719 (34.65)	747 (36.14)	820 (38.74)	0.001
Yes	3,684 (55.44)	1,356 (65.35)	1,320 (63.86)	1,307 (61.45)	
**Shortage of Nursing Staff**					
No	5,089 (80.53)	1,745 (87.56)	1,697 (85.58)	1,708 (85.19)	0.001
Yes	1,230 (19.47)	248 (12.44)	286 (14.42)	297 (14.81)	0.001
**Shortage of Clinical Staff**					
No	6,153 (97.37)	1,933 (96.99)	1,943 (97.98)	1,953 (97.41)	0.33
Yes	166 (2.63)	60 (3.01)	40 (2.02)	52 (2.59)	0.33
**Shortage of Aides**					
No	4,835 (76.52)	1,701 (85.35)	1,666 (84.01)	1,681 (83.84)	0.001
Yes	1,484 (23.48)	292 (14.65)	317 (15.99)	324 (16.16)	0.001
**Shortage of Other Staff**					
No	5,568 (88.13)	1,828 (91.72)	1,831 (92.33)	1,839 (91.72)	0.001
Yes	750 (11.87)	165 (8.28)	152 (7.67)	166 (8.28)	0.001
Total Beds (%)	87.88 (44.17)	132.97 (68.27)	127.67 (59.68)	134.97 (76.07)	0.001

*Source: Author's own analysis of study datasets of CMS Nursing Home COVID-19 Public File as of October 25, 2020, Brown University's LTCFocus, and Robert Graham Center's Social Deprivation Index*.

* *Minorities were classified as Black/Hispanic residents*.

*For continuous variables, t-tests were utilized. For categorical variables, chi-squares were utilized*.

Negative binomial regression results with Incidence Rate Ratios (IRR) are presented in Table 2. Model 1, after controlling for size and state fixed effects, shows that compared to nursing homes with no minorities, high-, medium-, and low-minority nursing homes had 66, 54, and 42% higher COVID-19 deaths, respectively (*p* < 0.001). In model 2, once facility level and resident characteristics were added, the risk of COVID-19 mortality in high-, medium-, and low-minority nursing homes is of 80, 62, and 42%, respectively, compared to facilities with no minorities (*p* < 0.001). In addition, Model 2 shows that nursing homes with a higher proportion of older residents and hypertension had greater COVID-19 mortality (*p* < 0.001). Additionally, nursing homes with a higher proportion of residents with congestive heart failure, obesity, and worse acuity were at greater risk of COVID-19 mortality (*p* < 0.05). Resident gender at the facility-level was not significantly related to COVID-19 deaths.

**Table 2:** Negative binomial regression results for incidence rate ratios (IRR) of COVID-19 deaths in nursing homes.

	**Model 1 (*****n*** **=** **12,761)**			**Model 2 (*****n*** **=** **12,483)**			**Model 3 (*****n*** **=** **12,461)**			**Model 4 (*****n*** **=** **11,178)**		
	**IRR**	**95% CI**		**IRR**	**95% CI**		**IRR**	**95% CI**		**IRR**	**95% CI**	
**Racial/ethnic minority residents^+^^,^^++^ (Ref. No minorities)**
Low (1–13.3%)	1.420^***^	1.286	1.568	1.421^***^	1.285	1.570	1.340^***^	1.208	1.487	1.348^***^	1.206	1.506
Medium (>13.3%–**≤** 30.3%)	1.538^***^	1.387	1.707	1.619^***^	1.453	1.803	1.459^***^	1.305	1.631	1.528^***^	1.355	1.723
High (>30.3%)	1.661^***^	1.496	1.844	1.804^***^	1.607	2.024	1.516^***^	1.338	1.717	1.611^***^	1.408	1.842
Total Beds	1.009^***^	1.008	1.010	1.009^***^	1.008	1.009	1.008^***^	1.008	1.009	1.008^***^	1.008	1.009
**Resident characteristics**												
Female residents (%)				1.001	0.997	1.004	0.999	0.995	1.003	1.000	0.996	1.004
Residents 65 + (%)				1.005^***^	1.002	1.008	1.007^***^	1.004	1.011	1.008^***^	1.004	1.011
Residents with Congestive Heart Failure (%)				1.004^*^	1.001	1.007	1.003	0.999	1.006	1.004^**^	1.000	1.007
Residents with Hypertension (%)				1.005^***^	1.001	1.008	1.005^***^	1.001	1.008	1.003^*^	1.000	1.007
Residents who are Obese (%)				1.003^*^	1.000	1.007	1.004^*^	1.001	1.007	1.004^**^	1.000	1.007
Acuity index				1.027^*^	0.997	1.058	1.024	0.994	1.055	1.028^*^	0.997	1.061
**Resource availability**												
Occupancy Rate							1.011^***^	1.008	1.014	1.012^***^	1.009	1.015
Medicaid share (%)							1.003^**^	1.001	1.005	1.001	0.999	1.004
Medicare share (%)							1.000	0.996	1.003	0.999	0.995	1.003
Social Deprivation Index							1.003^***^	1.001	1.004	1.002^***^	1.001	1.004
Non-Metro (Ref. Metro)							0.745^***^	0.685	0.811	0.770^***^	0.704	0.842
**Organizational characteristics**												
For-Profit Status (Ref. Not-For-Profit)										1.208^***^	1.105	1.319
Chain Affiliation (Ref. Independent)										1.067	0.988	1.153
Shortage of Nursing Staff										1.156^*^	1.000	1.336
Shortage of Clinical Staff										1.069	0.841	1.360
Shortage of Aides										1.005	0.877	1.151
Shortage of Other Aides										1.138^*^	0.984	1.317
Pseudo-*R*^2^	0.036			0.036			0.038			0.042		
Likelihood Ratio Test for Nested Models				1, 240.48^***^			163.34^***^			7, 014.27^***^		
Akaike Information Criterion (AIC)	55, 393.64			54, 631.94			54, 396.89			48, 308.75		
Bayesian Information Criterion (BIC)	55, 796.16			55, 077.87			54, 879.87			48, 828.59		

*Source: Author's own analysis of study datasets of CMS Nursing Home COVID-19 Public File as of October 25, 2020, Brown University's LTCFocus, and Robert Graham Center's Social Deprivation Index*.

* *p < 0.05*,

** *p < 0.01*,

*** *p < 0.001*.

+ *Minorities were classified as Black/Hispanic residents*.

++ * Post hoc tests showed that low-minority nursing homes were significantly different from medium-minority facilities in Model 2 only (p < 0.05). Low-minority nursing homes were significantly different from high-minority facilities in Models 1, 2 and 4 (p-value < 0.05). Medium-minority and high-minority facilities were not significantly different in any of the models (p-value < 0.05)*.

*Model 1: Controlled for size (beds) and interstate differences. Model 2: Variables from model 1, in addition to facility-level resident characteristics. Model 3: Variables from model 2, in addition to resource availability variables. Model 4: Variables from model 3, in addition to organizational characteristics*.

In Model 3, included resource availability at the nursing home and community levels, high-, medium-, and low-minority nursing homes had 52, 46, and 34% greater COVID-19 mortality (*p* < 0.001), respectively, compared to no-minority nursing homes. Every 10% in Medicaid census increases the COVID-19 deaths by 3% (*p* < 0.01). Facilities in more social deprived communities were at greater risk (*p* < 0.001) for COVID-19 mortality. On the other hand, nursing homes located in non-metro areas (*p* < 0.001) were at lower risk of COVID-19 deaths as compared to those in metro areas. During the initial wave of the coronavirus pandemic, urban centers were the hardest hit. Finally, for every 10% increase in occupancy, COVID-19 mortality increased by 11% (*p* < 0.001). This may be attributed to the fact that the virus may spread to more residents in more densely populated facilities.

In Model 4, after including other organizational factors, high-, medium-, and high-minority nursing homes had 61, 53, and 35% greater COVID-19 mortality (*p* < 0.001), respectively, compared to nursing homes with no minorities. For-profit nursing homes had 21% more COVID-19 mortality (*p* < 0.001). A shortage of nurse staff increased the risk of COVID-19 mortality by 21% (*p* < 0.001), and shortage of other aides increased the risk by 14% (*p* < 0.05). *Post hoc* comparisons of low-, medium-, and high-minority nursing homes IRRs in each model are included in Table 2.

**Figure 2:**
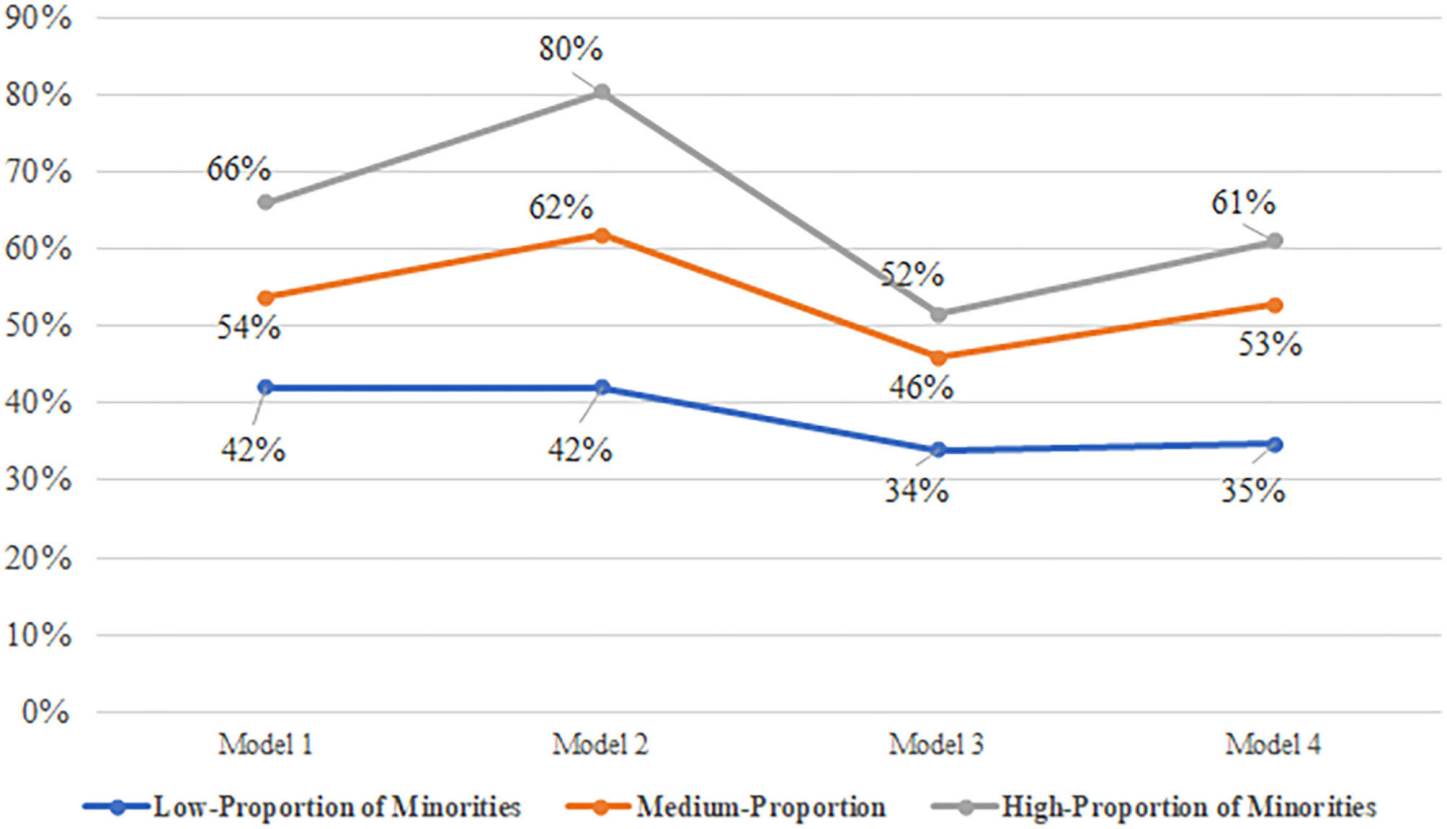
Incidence rate ratios of COVID-19 mortality by resident racial/ethnic mix (*n* = 12,914). CMS nursing home COVID-19 public file, brown University's LTCFocus, and Robert graham center's social deprivation index. No minorities is reference group; Low-proportion of minorities (0 < minority > 13.3 of Blacks/Hispanics); Medium-proportion of minorities (13.3 < minority < 30.3 of Blacks/Hispanics); High-proportion of minorities (30.3 < minority). Incidence rate ratios calculated from the negative binomial regression averaging over the remaining covariates. Model 1: Controlled for interstate differences and size. Model 2: Variables from model 1,and including facility-level resident characteristics. Model 3: Variables from model 2, and including resource availability. Model 4: Variables from model 3, and including organizational characteristics.

In summary, even after accounting for resource availability and other organizational characteristics, and controlling for facility-level resident characteristics and interstate differences, high-minority nursing homes had 61% greater COVID-19 deaths compared to those with no minorities (Figure 2).

## Discussion

This study found that nursing homes with high-minority populations were at the highest risk of COVID-19 deaths, even after accounting for resource availability, and other organizational factors, and controlling for resident characteristics. These findings belie an ugly but prominent truth, that the existing racial/ethnic disparities have real and tangible negative outcomes. These findings are consistent with prior studies showing that that Black and Hispanic nursing home residents are more likely than their White peers to reside in nursing homes characterized by inadequate resources, less staffing, higher deficiencies, poorer performance, and worse quality of care (5, 19). Further, these findings suggest that the nursing home industry continues to operate as a two-tier system based on race/ethnicity and socioeconomic status (6, 20).

Disparities observed in nursing homes may be a reflection of wider disparities observed in the incidence of COVID-19 among minority communities. One of the most telling findings was the issue of resource availability; nursing homes with higher levels of minorities were located in poorer, urban communities. Race/ethnicity has to be examined in the larger context of the social determinants of health (21). Resource availability is lacking in communities where many Black and Hispanic people reside, which ultimately impacts one's health (22). Nursing homes located in poorer, urban communities that serve more racial/ethnic minorities may face increased challenges in the delivery of high-quality care. As such, federal and state level policymakers should provide additional resources to these vulnerable nursing homes to help offset the cost of quality measures that will help reduce the risk of viral transmission. Such strategies could include disproportionate payments as in the case of hospitals. The allocation of financial resources to these nursing homes may be one way to provide these organizations with the additional support that they need.

However, even after accounting for resource availability and other organizational factors in this analysis, there were nursing home racial/ethnic disparities in COVID-19 deaths. This suggests the existence of systemic racial/ethnic inequalities. Minority populations, such as, Blacks and Hispanics, tend to have fewer alternatives for high quality nursing home care relative to Whites (19). Nursing home care is often geographically constrained to a certain community or concentrated group of individuals (9). The delivery of high-quality nursing home care is not equitable. Nursing homes remain relatively segregated, roughly mirroring the residential segregation within a community (20). The issue of de facto racial segregation of health care facilities has been notated by researchers and advocates (8, 23). From a policy perspective, nursing homes that serve high minority populations, may need additional resources, such as, funding for staffing and personal protective equipment. COVID-19 has sharpened the focus on structural and societal inequalities that have long existed.

This study also found that nursing homes reporting greater staffing shortages had worse mortality outcomes. Nursing homes with reported shortages should be monitored and offered additional assistance provided in the form of educational, safety guidelines, and staff. The scope of this pandemic is something that most health care facilities were not adequately prepared for. Nursing homes may need additional resources to get the necessary additional training on the recommend guidelines and procedures for infections and disease control. Beyond these measures, nursing homes may also need to train staff on how to communicate with residents (and each other) effectively and affectively, as to facilitate more productive communication but to also ease tensions and uncertainty, especially among residents with cognitive impairments and/or dementia (24). Furthermore, nursing homes have to educate and train their staff on the importance of personal protective equipment, active screening, social distancing, and how to effectively identify and treat residents who have been exposed (25). There is a need to provide information to the residents and staff about COVID-19, along with the warning signs and provide active screenings. From a process standpoint, this includes wearing gowns, gloves, facemask, and eye protection; however, this may be challenging in nursing homes where there are limited resources. Policymakers may need to intervene to ensure proper protection equipment is available for all nursing home facilities. Nursing homes must reinforce adherence to infection prevention and control measures, including hand hygiene and selection and use of personal protective equipment (26). Healthcare leaders have to use this crisis as an opportunity to learn and grow in order to be better prepared for the future.

There are some limitations in this study that should be noted. First, the CMS Nursing Home COVID-19 Public File is a dataset that is revised weekly, and the data was as of October 25, 2020. Due to the rapidly changing nature of this pandemic, the data may not reflect the current environment. Second, there were limitations due to the availability of data especially as it related to resident level comorbidities. Resident health data was extracted from LTCFocus and capture some of the health issues that may lead to greatest complication due to COVID-19. Third, potential undertesting at the beginning of the pandemic may have resulted in underreported COVID-19 deaths (27). Furthermore, the COVID-19 mortality data are reported by nursing homes to the CDC, and may be subject to inaccuracies. However, both CMS and CDC ensure the accuracy of the reported numbers by performing data quality checks.

Despite these limitations, this study sheds light into the existing racial/ethnic disparities in COVID-19 deaths. These findings underscore prior research showing that the nursing home industry operate on a two-tier system based on race/ethnicity and socioeconomic status. Policy interventions are needed to address some of the resource allocation and systemic racial/ethnic inequality issues at the core of these disparities.

## Data Availability Statement

The raw data supporting the conclusions of this article will be made available by the authors, without undue reservation.

## Author Contributions

RW-M, GD, and JL: conception and design. RW-M: administrative support. GD and GO: provision of study materials or patients. GD and AG: collection and assembly of data. GD: data analysis and interpretation. JL and RW-M: manuscript writing. All authors contributed to the article and approved the submitted version.

## Conflict of Interest

The authors declare that the research was conducted in the absence of any commercial or financial relationships that could be construed as a potential conflict of interest.

## References

[1] KFF. State Data and Policy Actions to Address Coronavirus. Kaiser Family Foundation. Available online at: https://www.kff.org/health-costs/issue-brief/state-data-and-policy-actions-to-address-coronavirus/ (accessed July 17, 2020).

[2] Chowkwanyun M Reed Jr AL. Racial health disparities and COVID-19—caution and context. N Engl J Med. (2020) 383:2010–3. 10.1056/NEJMp2012910 32374952

[3] Feng Z Fennell ML Tyler DA Clark M Mor V. Growth of racial and ethnic minorities in US nursing homes driven by demographics and possible disparities in options. Health Affairs. (2011) 30:1358–65. 10.1377/hlthaff.2011.0126 21734211 PMC3785292

[4] Li Y Cai X. Racial and ethnic disparities in social engagement among US nursing home residents. Med Care. (2014) 52:314. 10.1097/MLR.0000000000000088 24848205 PMC4031618

[5] Chisholm L Weech-Maldonado R Laberge A Lin FC Hyer K. Nursing home quality and financial performance: does the racial composition of residents matter? Health Serv Res. (2013) 48 :2060–80. 10.1111/1475-6773.12079 23800123 PMC3805666

[6] Mor V Zinn J Angelelli J Teno JM Miller SC. Driven to tiers: socioeconomic and racial disparities in the quality of nursing home care. Milbank Q. (2004) 82:227–56. 10.1111/j.0887-378X.2004.00309.x 15225329 PMC2690171

[7] Li Y Yin J Cai X Temkin-Greener H Mukamel DB. Association of race and sites of care with pressure ulcers in high-risk nursing home residents. JAMA. (2011) 306:179–86. 10.1001/jama.2011.942 21750295 PMC4108174

[8] Institute of Medicine. Unequal treatment: Confronting Racial and Ethnic Disparities in Health Care. Washington, DC: The National Academies Press (2002). 25032386

[9] Tamara Konetzka R Grabowski DC Perraillon MC Werner RM. Nursing home 5-star rating system exacerbates disparities in quality, by payer source. Health affairs. (2015) 34:819–27. 10.1377/hlthaff.2014.1084 25941284 PMC6344885

[10] Li Y Cen X Cai X Temkin-Greener H. Racial and ethnic disparities in COVID-19 infections and deaths across US nursing homes. J Am Geriatr Soc. (2020) 68:2454–61. 10.1111/jgs.16847 32955105 PMC7537079

[11] The Centers for Medicare and Medicaid Services. COVID-19 Nursing Home Data. 6th ed. Baltimore, MD (2020).

[12] Abrams HR Loomer L Gandhi A Grabowski DC. Characteristics of US nursing homes with COVID-19 cases. J Am Geriatr Soc. (2020) 68:1653–56. 10.1111/jgs.16661 32484912 PMC7300642

[13] Gorges RJ Konetzka RT. Staffing levels and COVID-19 cases and outbreaks in US nursing homes. J Am Geriatr Soc. (2020) 68:2462–6. 10.1111/jgs.16787 32770832 PMC7436613

[14] Ouslander JG Grabowski DC. COVID-19 in nursing homes: calming the perfect storm. J Am GeriatrSoc. (2020) 68:2153–62. 10.1111/jgs.16784 32735036

[15] Pfeffer J Salancik GR. The External Control of Organizations: A Resource Dependence Perspective. Redwood City, CA: Stanford University Press (2003).

[16] Hefele JG Ritter GA Bishop CE Acevedo A Ramos C Nsiah-Jefferson LA. Examining racial and ethnic differences in nursing home quality. Jt Comm J Qual Patient Saf. (2017) 43:554–64. 10.1016/j.jcjq.2017.06.003 29056175

[17] Butler DC Petterson S Phillips RL Bazemore AW. Measures of social deprivation that predict health care access and need within a rational area of primary care service delivery. Health Serv Res. (2013) 48 :539–59. 10.1111/j.1475-6773.2012.01449.x 22816561 PMC3626349

[18] Shrestha N. Detecting multicollinearity in regression analysis. Am J Appl Math Stat. (2020) 8:39–42. 10.12691/ajams-8-2-1

[19] Fennell ML Feng Z Clark MA Mor V. Elderly Hispanics more likely to reside in poor-quality nursing homes. Health Affairs. (2010) 29:65–73. 10.1377/hlthaff.2009.0003 20048362 PMC3825737

[20] Smith DB Feng Z Fennell ML Zinn JS Mor V. Separate and unequal: racial segregation and disparities in quality across US nursing homes. Health Affairs. (2007) 26:1448–58. 10.1377/hlthaff.26.5.1448 17848457

[21] Harding K. The Rabbit Effect: Live Longer, Happier, and Healthier With the Groundbreaking Science of Kindness. New York, NY: Simon and Schuster (2019).

[22] Yancy CW. COVID-19 and African Americans. JAMA. (2020) 323:1891–92. 10.1001/jama.2020.6548 32293639

[23] U.S. Civil Rights Commission. The Federal Civil Rights Enforcement Effort: One Year Later. Washington, DC: Government Printing Office (1971).

[24] Levy-Storms L. Therapeutic communication training in long-term care institutions: recommendations for future research. Patient Educ Couns. (2008) 73:8–21. 10.1016/j.pec.2008.05.026 18656320

[25] Dwolatzky T. If not now, when? The role of geriatric leadership as Covid-19 brings the world to its knees. Front Med. (2020) 7:232. 10.3389/fmed.2020.00232 32574329 PMC7243654

[26] COVID TC Team R. Severe outcomes among patients with Coronavirus disease 2019 (COVID-19)-United States, February 12-March 16, 2020. MMWR Morb Mortal Wkly Rep. (2020) 69:343–6. 10.15585/mmwr.mm6912e2 32214079 PMC7725513

[27] Lin TP Wan KH Huang SS Jonas JB Hui DS Lam DS. Death tolls of COVID-19: where come the fallacies and ways to make them more accurate. Global Public Health. (2020) 15:1582–7. 10.1080/17441692.2020.1808040 32787510

